# Nurses' Help and Self-Help in Managing Emotions While Working With Elderly Patients Ill With Chronic Cardiovascular Diseases and Experiencing Fear of Death

**DOI:** 10.1155/nrp/5060286

**Published:** 2025-11-29

**Authors:** Vilma Zydziunaite, Kristina Bulkina

**Affiliations:** Department of Nursing and Social Welfare, Faculty of Health Sciences, Klaipedos Valstybinė Kolegija/Higher Education Institution, Klaipėda, Lithuania

**Keywords:** chronic cardiovascular disease, elderly patient, fear of death, helping, managing emotions, old patient, self-help

## Abstract

**Background:**

Advances in health care seem incapable of eliminating fear of death among elderly patients (Eps) ill with chronic cardiovascular diseases (CVD). Lack of research on the topic shows the uniqueness and novelty of this study. The following research questions were raised: “How do nurses assist elderly patients ill with chronic CVD who experience fear of death? What kind of emotions do nurses experience and how do they deal with this while working with elderly patients ill with chronic CVD who experience fear of death?”

**Aim:**

The study aimed to highlight how nurses help patients, and self-help, in managing personal emotions while working with elderly patients ill with chronic CVD and experiencing fear of death.

**Setting:**

The research setting in this study was five hospitals from the five biggest cities of Lithuania.

**Methods:**

In the research study, a qualitative research design was applied. Fifteen individual semi-structured interviews were conducted with nurses. For data analysis, latent qualitative content analysis was used.

**Results:**

Findings showed that nurses assist elderly patients ill with chronic CVD and their families by helping overcome their fear of death by using empathetic and respectful communication, and cooperating with other specialists for managing and supporting patients' needs. Nurses help themselves by managing their emotions through maintaining professional distance and demonstrating professionalism and confidentiality.

**Conclusions:**

Nurses assist elderly patients ill with chronic CVD who experience fear of death to manage their emotions by communicating respectfully and empathetically, creating dignified relationships, establishing a comfortable emotional and social environment, providing specific information, recommending therapy for emotional well-being, organizing special professional assistance, and encouraging family involvement. Nurses help themselves while managing their own emotions by reflecting in- and after-acting at work, detaching emotions from work, and finding time for personal interests.

## 1. Introduction

Every country worldwide is experiencing growth in the number of elderly individuals. By 2030, one in six people in the world will be aged 60 years or above. The number of persons aged 80 years or older is expected to triple between 2020 and 2050 to reach 426 million [1, 2]. While developed countries today have the highest share of older persons, developing countries are witnessing a rapid rate of population aging, leaving many ill-prepared for the new realities. The elderly population is that share of the population aged 65 years and above, known as “dependent,” one that that does not work but relies on others for the goods and services its members consume [3–6].

The global shift in health care has led to a long-term care context that has become the place of care for elderly patients [4, 5]. Nurses have the most contact time with patients; they should not only be familiar with the characteristics of elderly patients ill with chronic cardiovascular diseases (CVD) but also acquire knowledge of the social, moral, and ethical aspects of nursing [6, 7]. In the nursing of elderly patients ill with CVD, help, support, and assistance to patients and their families is a primary focus and concern [7, 8]. CVD and cancer together account for nearly 46% of all deaths among older individuals [1–3].

Advances in health care seem incapable of eliminating fear of death among elderly patients ill with CVD. Fear of death is a feeling that causes anxiety, sadness, poor sleep, lack of appetite, and emotional sensitivity in elderly patients, hence patients need more attention from nurses. Elderly patients ill with CVD express the need to talk a lot and be listened to; they ask nurses various questions and require detailed answers, especially about the changes in the physiology of the body [8, 9]. Nurses should actively communicate with elderly patients ill with CVD, establish a relationship of mutual trust and understanding; and carry out targeted psychological caring, illness management, dietary prescriptions, and discharge guidance according to elderly patients' different conditions and personality characteristics, so as to improve the quality of nursing and the recovery rate of elderly patients ill with CVD [9, 10].

Nurses have more contact with such elderly patients than any other health care professionals. They get to know the elderly patients and their families well. The more often nurses meet the elderly patients ill with CVD, the greater the bond they develop with their patients. Compassion, sadness, and helplessness are the most common emotions experienced by nurses while working with elderly patients ill with CVD who have fear of death, regardless of the nurses' length of service [10, 11]. Nurses, while caring for elderly patients ill with CVD who experience fear of death, feel stressed [11, 12]. Nurses must use their emotional, psychological, and moral resources when communicating with elderly patients. This requires empathy, sensitivity, compassion, goodwill, openness, patience, and tolerance [12, 13]. These qualities positively affect the emotions, mood, hope, and positivity of elderly patients ill with CVD, and this is the core factor influencing the highest quality nursing of such patients [13, 14]. However, the long-term health of elderly patients ill with CVD who experience fear of death depletes the emotional and mental health of nurses, so self-help, that is, the ability to help themselves while working in such a sensitive nursing environment requires great dedication to patients [14, 15].

Nursing research of elderly patients with chronic heart diseases focuses on physiological and psychological changes, mental disorders, the role of nurses, the dying process, and death anxiety [15–22]. However, only one specific study [22, 23] on elderly patients ill with CVD and experiencing fear of death was found while searching the Medline, Ovid Medline, and Pubmed international databases for the past two decades. Researchers in this study revealed that changes of emotions, behavior, and physiological symptoms of elderly patients ill with CCVDs who experience fear of death are related to their psychological state. The multiple needs of elderly patients are intensified while they experience fear of death.

Lack of research on the topic related to elderly patients ill with CVD who experience fear of death shows the uniqueness and novelty of this study. So, the following research questions for this study were raised: “How do nurses assist elderly patients ill with chronic cardiovascular diseases who experience fear of death? What kind of emotions do nurses experience and how do they deal with this while working with elderly patients ill with chronic cardiovascular diseases who experience fear of death?”

The novelty of this research lies in its specific focus on the intersection of chronic CVD, fear of death, and the emotional labor of nurses, examining both external (help) and internal (self-help) coping strategies. While existing research often covers general nursing stress or emotional intelligence, this specific study bridges the gap by linking the unique emotional challenges nurses face with these particular patient groups and exploring the specific emotional management tools they use to cope. The research examines both the help nurses receive from others (peer support, etc.) and the self-help strategies they employ (self-efficacy, emotional intelligence) to manage their own emotions. The study identifies the emotional, behavioral, and physical signs that nurses observe to recognize when a patient is experiencing a fear of death, which is a key practical, experience-based insight. By analyzing nurses' attitudes and coping mechanisms, the research aims to inform the development of targeted interventions to support nurses, which is a crucial next step in improving their emotional well-being and quality of patient care.

## 2. Aim

The following aim was formed for the study: To reveal how nurses help patients, and self-help in managing personal emotions, while working with elderly patients ill with chronic CVD and experiencing fear of death.

## 3. Methods

### 3.1. Study Design

In the research study, a qualitative research design was applied. It is useful in increasing nurses' understanding of their own and patients' experiences by allowing for effective and efficient interventions in caring, and the anticipation of issues that might be encountered by the patient in a specific context [22–24].

### 3.2. Sample

Nurses who work with elderly patients ill with CVD in hospitals from five biggest cities of Lithuania (Kaunas, Klaipėda, Panevėžys, Šiauliai, and Vilnius) and three nurses from each city participated in the research. Purposive sampling was applied in the research study: research participants were deliberately selected because they are characterized by features that researchers needed in the sample. The following criteria were used for selecting informants: at least 10 years' nursing experience with elderly patients ill with CVD; at least 10 years' work experience in a health care institution; and a Bachelor's degree in nursing acquired from a higher education institution. A total of 15 nurse practitioners, all women, participated in the research (see Table 1).

The sample size was determined by data saturation, when in the last interview conducted, all the information collected so far was repeated and no new details emerged, which means that enough data were collected in order to conclude the research findings and additional data collection did not produce further valuable information for the study. To perform an interview saturation analysis, researchers conducted interviews, analyzed them one by one, and identified when new data stopped providing new insights and categories. This involved an iterative process of coding and tracking the emergence of categories, comparing new data against existing findings, and adjusting the sample size as needed [24]. Data saturation in the selection of this study participants meant that data were collected until no new aspects were found in the interview text. Therefore, the data were analyzed immediately after the interview was conducted and its text was transcribed, and after conducting the initial analysis— reflecting, forming open codes, and focusing on emerging key concepts. This was done by using purposeful sampling, interviewing until researchers have a sufficient range of perspectives, and then analyzing transcripts to count new codes in a series of consecutive interviews. Once several interviews in a row yielded no new codes, or new codes fall below a predetermined threshold, researchers stopped collecting data [25]. In this study, data saturation was determined at the 13th interview, when new codes did not appear. However, two additional interviews were conducted to ensure that there was no doubt about the validity of data saturation.

### 3.3. Data Collection

Data were collected between February 2023 and August 2024 by using semi-structured interviews. Interviews with research participants were conducted away from workplaces and before or after nurses' working hours. The date and time of the interviews with each study participant were agreed upon individually. Before starting the interview, the aim, objectives, and the research value for nursing practice were described to each interview participant individually. All interviews were recorded, transcribed, and analyzed consistently one after the other so that new details were not missed. A total of 15 interviews were conducted. Interviews RN1–RN8 were conducted online, while interviews RN9–RN15 were conducted in-person. The interview with each nurse ranged from 50 to 182 min.

### 3.4. Tool

The semi-structured interview questionnaire used in interview consisted of demographic and content parts. In the demographic part, nurses were asked for information about their age, length of practice in health care and work with elderly patients ill with CVD, and their highest level of education in nursing. The content part consisted of four questions: (i) How do you help elderly patients ill with CVD who experience the fear of death? (ii) What kind of support do you provide to elderly patients ill with CVD who experience the fear of death? (iii) What kind of emotions do you experience while working with elderly patients ill with CVD who experience the fear of death? (iv) How do you manage emotions while working with elderly patients ill with CVD who experience the fear of death? In each case, participants were asked to share their experiences, talk about cases they had encountered, decisions they made, and the meanings of their experiences. During each interview, follow-up questions were also asked, which were specific in each case. However, the thoughts expressed by the research participants were always explored in depth, and terms, expressions, or metaphors that were unclear to the researchers were not left unexplained. In such cases, participants were asked to explain in more detail what this meant and to expand on these points, focusing on specific contexts relevant to the interviewers.

### 3.5. Data Analysis

In the research, the latent type of qualitative content analysis was used for data analysis. Latent content analysis looks at the underlying aspects of the phenomenon under research [24]. This method provides the opportunity for researchers to examine the content of nurses' experiences while working with elderly patients ill with CVD who experience fear of death. This analytic method is focused on identifying, enumerating, and recording the emergence and accentuation of particular notions, words, and phrases [25]. The specificity of coding steps in latent qualitative content analysis involved moving beyond the surface level of the data to interpret underlying meanings through a process of abstraction and interpretation. Key steps included developing a coding scheme with clear definitions and rules, applying codes to the data, and iteratively refining codes and categories based on a deep understanding of the text and your research question. This approach required constant comparison and an awareness of how the researcher's perspective influences the analysis [26].

The coding was conducted via detailed steps [23–25]:

1. Reading and familiarizing. Reading all of the data in order to become familiar with it. This step helped researchers to think holistically.
2. Applying codes. Researchers were going through the data, identifying meaning units, and applying the defined codes. At this stage, researchers remained close to the original text while the latent analysis began to interpret the deeper meaning.
3. First-round coding. Identifying the “big picture” of meaning units through comprehending. This involved organizing the data by identifying and labelling sections of the interview texts according to their “big-picture meaning.”
4. Second-round coding. Developing subcategories and fine-grained codes by taking a closer look at the text within each of the sections that were coded as a particular big-picture category, choosing a big-picture category, and looking at all of the sections in all of the transcripts under that category.
5. Refining the fine-grained subcategories. After coding all the text within the big-picture categories into subcategories, all the fine-grained subcategories were compared and refined. It was found that some subcategories were so similar that they were integrated into one; others were different from each other and remained as autonomous subcategories. Also considered was whether any subcategories were related to a different big-picture category. This would require comparison of all the meaning units of text coded in each subcategory from all transcripts of interviews. In carrying this comparison, it was realized that some categories were still too broad, or too vague, which necessitated the need for specification.
6. Synthesizing and interpreting. Trying to stay closer to the research phenomenon under study and producing an interpretation that provides a rich answer to the research question. This is a process of connecting the categories to create a narrative that gives an overall explanation of the research phenomenon.
7. Writing memos. Researchers documented their impressions, reflections, and analytical thoughts during the coding process. Memos helped to develop their understandings of the codes and their connections to the research question.
8. Handling data transparently. Researchers were clear about what is and is not coded. Researchers assured that their selection criteria were well-defined and transparent in their reporting.

### 3.6. Trustworthiness

The trustworthiness of findings was ensured through [25]: credibility (researchers were involved in all procedures regarding data collection, analysis, and interpretation); transferability (researchers worked with data analysis autonomously, then shared the data analyses among themselves for cross-checking, detailed explanations, and consensus-making); dependability (both authors kept the academic rigor through comprehensively documented processes of data collection, analysis, and interpretation); and confirmability (authors systematically organized their online meetings for peer debriefing, member checking, and reflecting the particular study phases and steps).

Reflexivity included the researchers' critical self-reflection on how their own biases, values, and experiences might influence the research process, including data collection and analysis. It contributed to trustworthiness by ensuring a more rigorous and transparent study, making the findings more credible and ethically sound. By acknowledging and documenting their subjectivity, researchers understood and managed their impact, leading to better data interpretation and more reliable findings. The researcher's role was intertwined with the data they collected and interpreted. By being reflexive, researchers understood how their presence might affect participant responses or how their perspectives might color the analysis, improving the quality and integrity of the findings. By critically examining their own role, researchers increased the transparency of their study. This self-awareness made the research process more understandable and trustworthy for others [26].

### 3.7. Ethical Considerations

The study was conducted according to the guidelines of the Declaration of Helsinki, and approved by the Ethics Committee of Klaipėda State University of Applied Sciences/KVK (KVK-TE-1-2023/01/30) and the Ethics Committee of Klaipeda University, Faculty of Health Sciences, Department of Nursing (SvMF-SlK-EK-1-2023/02/08).

The following principles were the basis for the study [24, 25]: (i) Informed consent (informants were informed about the research purpose and objectives, procedures of data collection and its application). (ii) Confidentiality and privacy (participants' individual information and interview transcriptions were kept confidentially in separate computer files with codes. For keeping confidentiality, pseudonyms were used. All the informants had the opportunity to refuse to answer questions or to share the particular information). (iii) Respect for participants (researchers were aware of their own biases and assumptions in avoiding influence of their personal attitudes on interview participants. Researchers were sensitive and careful to conduct the study in a respectful manner. The findings were used in a respectful manner to ensure that they benefit the nurses' professional community and the patients).

## 4. Results

From data analysis emerged four categories that describe the help for patients and nurses' self-help in managing emotions while working with elderly patients ill with CVD and who experience fear of death: assisting through help, providing support, living with emotions, and managing one's own emotions.

### 4.1. Category: Help-Based Assisting

Nurses help elderly patients overcome their fear of death by using empathetic communication, providing patients with information, and applying therapeutic measures prescribed by the physician. Empathetic and respectful communication is essential for patients who feel afraid of death, so nurses try to reassure them by talking about topical issues and expressing understanding: “Respectful relationships are established, a comfortable environment is created for expressing feelings” (RN12); “I take into account the individual needs of patients during communication” (RN14). Providing information is important for elderly patients who are ill with CVD and experience fear of death (see Table 2). Therefore, nurses provide them with information about the course of the disease and complications: “about the disease process or, if necessary, about medical terms and aspects of the disease, reducing their fear of the unknown” (RN11).

If necessary, nurses cooperate with other specialists, psychologists, in order to inform patients as accurately and in as much detail as possible. In order to alleviate the tension caused by the fear of death in patients, nurses use art and music as therapeutic tools, improving the emotional well-being of patients: “relaxation, breathing exercises to reduce anxiety, meditation are used” (RN5).

### 4.2. Category: Providing Support

Nurses provide support for patients through therapeutic recommendations that support emotional well-being, special assistance, and encourage family participation/involvement. Most often, elderly patients and their family members are given recommendations on participating in therapy sessions, choosing different types of therapy tools, artistic and creative activities, meditation, and spiritual preparation sessions to reduce their stress levels. If the elderly patients' tendency toward creative activities is observed, they are “encouraged to engage in creative or artistic activities, to express their feelings through art” (RN6). Recommendations are also given to elderly patients' family members regarding the use of alternative therapy methods, breathing exercises, and participation in meditation (see Table 3).

Elderly patients are recommended to consult with various specialists, for example, “the patient is advised to contact a psychologist or therapist who can help understand and overcome the fear of death” (RN3). Nurses advise elderly patients to discuss their questions with physicians and specialists at the health care institution, since nurses cannot answer all questions and provide the necessary information. Nurses believe that the participation of the elderly patients' family members in the nursing process is very important, therefore the aim is to “involve family members in the nursing process” (RN11).

Nurses advise family members to spend more time with elderly patients so that they do not feel lonely.

### 4.3. Category: Living With Emotions

The characteristic of nurses' experienced emotions while working with elderly patients ill with CVD who experience fear of death are pity, sympathy, concern, helplessness, anger, and doubt. They feel compassion, concern, and pity for the patient's health condition. Nurses experience concern for the elderly patients' well-being and want to provide them with the necessary assistance, therefore they “try to ensure constant communication, take care of the patient's needs” (RN6), “monitor symptoms and effectively collaborate with other health care professionals to ensure the optimal level of patient care” (RN7). Nurses experience a feeling of helplessness, “especially in cases where it is difficult to alleviate the patient's suffering” (RN8) (see Table 4).

Nurses sometimes doubt the appropriateness of elderly patients' functions, because in many cases they cannot provide the necessary assistance to patients and alleviate their suffering.

### 4.4. Category: Managing Own Emotions

Nurses manage their personal emotions while working with elderly patients ill with CVD who experience fear of death through maintaining professional distance (“so that the patient has space to express emotions, I would maintain clear boundaries between professional and personal relationships” RN11), professionalism, and confidentiality. Reflection is used to manage emotions by thinking about experienced emotions and assessing their impact on professional nursing activities (see Table 5).

Nurses learn to manage their emotions, devote time to hobbies, relax after working hours, rest, and spend leisure time with family and on interesting activities. When overcoming emotions, nurses focus on tasks and goals, thus distancing themselves from negative emotions and avoiding emotional burnout.

## 5. Discussion

Findings revealed how nurses assist, what kind of emotions they experience, and how they manage their own emotions while working with elderly patients ill with CVD who experience fear of death.

### 5.1. Nurses Help to Manage Their Emotions for Elderly Patients Ill With CVD Who Experience Fear of Death

In the research study, assisting of nurses incorporates caring attitude, support, and sensitivity which is related to compassion. Compassion is fundamental to palliative care and viewed as a cornerstone of high–quality care provision which includes information and dialogue and is a premise for creating a space for dying and family caregivers' acceptance of death [26, 27]. However, compassion is related to helping, as our study results show. Therefore, compassion and helping must be understood as complex phenomena because they are not habits or skills, and therefore their interpretation without a value context in nursing becomes problematic from a theoretical, methodological, and/or empirical point of view. Nurses assist elderly patients ill with CVD and their families by helping patients overcome their fear of death by using empathetic and respectful communication. These findings are strengthened by the results of studies which also state that support to patients and their relatives during their mourning and anxiety should be focused primarily on communication [19–21].

This research study provided evidence that nurses cooperate with other specialists, psychologists, in order to inform elderly patients ill with CVD who experience fear of death as accurately and in as much detail as possible. Interpersonal communication and information support every nursing activity [19, 20]. Health care professionals indicate that when they address elderly patients' problems together, seeing them from different professional perspectives, they are resolved more effectively, because professional perspectives complement each other [26, 27].

In order to alleviate the tension caused by fear of death in elderly patients, nurses use art and music as therapeutic tools, improving the emotional well-being of patients. Support for elderly patients and their relatives is provided not only through direct nursing actions but also through using artwork with the focus on cultural and spiritual care [12–15]. Nurses are committed to meeting the diverse and complex needs of patients with competence and compassion. Depending on the role and setting of the nurse's work, these include managing and supporting the needs of patients with complex needs, having emotional conversations with patients and families, and confronting challenging social and ethical issues [27–30].

The research study showed that nurses provide support for elderly patients ill with CVD who experience fear of death through therapeutic recommendations that support emotional well-being, special assistance, and encourage family participation/involvement. Nurses, while providing emotional support to patients and their families, are focused on warmth and kindness, deep listening, and social connection in the process of treatment [30–32]. Through nonverbal interventions and creative arts, therapy nurses can help elderly patients access, explore, and share authentic emotion in different forms. By reconstructing meaning through arts, patients and their family members may confront, reflect, and better cope with traumatic experiences while developing social support and deepening relations with their relatives and health care personnel [7–11, 13]. Research findings proved that elderly patients ill with CVD who experience fear of death and their family members are given recommendations on participating in therapy sessions, choosing different types of therapy tools, artistic and creative activities, meditation, and spiritual preparation sessions to reduce their stress. The spiritual aspect in nursing of elderly patients ill with CVD is related to greater recovery of illness, improved quality of life, and psychological support [14, 15, 18–20, 22]. Family involvement in nursing is a core aspect of providing patient and family-focused care and ensuring optimal patient outcomes [27, 31, 32]. Participation of the elderly patients' families in their nursing is very important. The elderly patients' relatives would like to be more closely involved, with the aim of providing maximum support to them. Nurses advise family members to spend more time with elderly patients ill with CVD who experience fear of death so that they do not feel lonely. Family members' talking and listening, and the adequate communication are protective factors for elderly patients with CVD moral distress and help to reduce misunderstandings [4, 5, 16, 28].

### 5.2. Emotions Experienced by Nurses While Working With Elderly Patients Ill With CVD Who Experience Fear of Death

The characteristics of nurses' emotions while working with elderly patients ill with CVD who experience fear of death are pity, sympathy, concern, helplessness, anger and doubt, compassion, concern, and pity for the patient's health condition, especially in cases where it is difficult to alleviate the patient's suffering. Nurses accept death as a natural phase of human life. Nurses feel sadness, helplessness, regret, and a high level of stress with strong emotions [9, 11, 13, 28, 31, 32] while observing elderly patients who experience fear of death. From research findings it is evident that nurses are concerned for the well-being of elderly patients ill with CCVDs and want to provide them with the necessary assistance. Therefore, they ensure constant communication and take care of their needs. Effective communication between nurses and patients is a key for the positive outcome of personalized nursing of patients. To achieve this, nurses must understand and help their patients by devoting time to their concerns [12, 21, 29, 31, 32].

### 5.3. Nurses' Self-Help in Managing Emotions While Working With Elderly Patients Ill With CVD Who Experience Fear of Death

Nurses manage their personal emotions while working with elderly patients ill with CVD by maintaining professional distance, professionalism, and confidentiality. So, nurses want to be of benefit to elderly patients and their families while struggling to balance being personal and professional. Reflection is used to manage emotions by thinking about experienced emotions and assessing their impact on professional nursing activities. Nurses' reflection is associated with nurses' work engagement and self-efficacy [10–12, 14, 20–22, 30, 31]. Nurses, while working with elderly patients ill with CVD who experience fear of death, learned to manage their own emotions, devoting time to hobbies and relaxation after working hours—rest, leisure time with family, and interesting activities. Enjoyable leisure activities, taken in the aggregate, are associated positively with nurses' psychosocial and physical health and well-being. Nurses perform specific roles in health care and often face stress, which is inseparable from work overload, conflicts, and various health care–related accidents [30, 31]. Resilience helps the nurses to reduce the consequences of stress [14]. Findings showed that when nurses overcome emotions, they focus on tasks and goals, thus distancing themselves from negative emotions and avoiding emotional burnout while working with elderly patients with CVD.

### 5.4. Added Value of Research Study

Research on nurses managing patients' emotions adds value to Watson's theory by emphasizing the importance of recognizing patients' signs of fear of death and by integrating nurses' self-help strategies into Watson's [33] theory framework in which care is the core of nursing and includes interpersonal attempts to enhance and maintain health, humanity, and well-being. The conducted research highlights how nurses can use the provision of a supportive, protective, and/or corrective mental, physical, societal, and spiritual environment factor to address both patient and nurse needs through emotional and psychological support, which can enhance the patient's self-care behaviors and the nurse's own ability to cope.

Research findings can inform nursing education, emotional support programs, and institutional policy by highlighting the need for training in emotional resilience and empathy, creating structured support systems for nurses, and fostering a supportive workplace culture. Specifically, education should include resilience and emotional intelligence training, support programs should offer resources like counseling and mentorship, and institutional policies must be revised to create a work environment that addresses the emotional toll of end-of-life care, such as by acknowledging staff feelings like compassion and sadness and establishing policies for compassionate care.

### 5.5. Study Limitations and Strengths

Key limitations of qualitative latent content analysis include the subjectivity because of researchers' interpretation, the time-intensive nature of the process, the potential for over-interpreting data, possibility of overlooking manifest content, and challenges with generalizability because of small sample size. As the analysis relied on interpretation of underlying, latent meanings, it was subjective and could lead to different conclusions from the same data set depending on the researchers' background or preconceptions. The data analysis process was time-consuming and required deep engagement with the data, often involving multiple readings and rereading of texts to identify subcategories and interpret categories. There was a risk of overinterpretation because researchers were able to focus so intensely on uncovering latent meanings that they could miss the more obvious manifest content or may infer meanings that are not fully supported by the data. The analysis was limited to the recorded content. Important “real-time” features, such as nonverbal communication, body language, and inflection, were not included in the analysis.

The transferability of research on nurses' management of emotions when caring for elderly patients with chronic CVD and fear of death is limited by the complexity of the issues, but key findings can be adapted. Core concepts like recognizing patient symptoms (emotional, behavioral, and physiological) and identifying the importance of self-help strategies (emotional coping, spiritual support, and competence) are likely transferable, but specific interventions may need tailoring to different contexts, as indicated by studies showing varying outcomes and support needs, such as the need for professional help or workplace discussions. Nurses can use identified emotional, behavioral, and physiological symptoms to recognize a patient's fear of death. This helps in providing appropriate care. Study highlights the importance of nurses' self-care and emotional coping mechanisms for managing stress and anxiety, which is a critical aspect of providing quality care. A patient's spirituality and a nurse's self-efficacy are important factors that influence their ability to provide and receive end-of-life care. Talking about death in the workplace and having support from colleagues can be a positive factor for nurses, although the type of support needed can vary.

## 6. Implications

This article reveals, through empirical evidence, the need to see and strengthen the emotional health of nurses not only when working with elderly patients ill with chronic CVD who experience fear of death but also for nurses working in other areas of nursing.

## 7. Conclusions

Nurses assist elderly patients ill with chronic CVD, and who experience fear of death, to manage their emotions through: communicating respectfully and empathetically; creating dignified relationships; establishing a comfortable environment emotionally and socially; providing specific information; recommending therapy for emotional well-being; organizing special professional assistance; and encouraging family involvement.

Nurses experience pity, sympathy, concern, helplessness, anger, doubt, and compassion for their elderly patients with chronic CVD. Nurses are living a professional life with their emotions by acting with compassionate mercy, worrying about their professional powerlessness in some cases, feeling internal moral outrage and suffering from doubts, while helping elderly patients ill with chronic CVD who fear death. Nurses are concerned about patients' well-being, and, while helping them to manage emotions, nurses provide patients with necessary assistance, ensure constant communication, and take care of their needs.

Nurses who work with such elderly patients manage their own emotions by maintaining a professional distance while communicating with patients and their families, reflecting both during and after work, detaching emotions from work, and making time for personal interests. These nurses maintain professionalism and confidentiality.

## Figures and Tables

**Table 1:** Demographic characteristics of research participants.

Code	Age (years old)	Highest level of education acquired	Total work experience (years) in health care	Work experience (years) with elderly patients ill with CDD
RN1	57	Bachelor of Nursing	35	18
RN2	44	Master of Nursing	22	20
RN3	47	Master of Nursing	25	25
RN4	42	Master of Nursing	20	15
RN5	49	Bachelor of Nursing	28	21
RN6	52	Bachelor of Nursing	30	30
RN7	56	Master of Nursing	35	24
RN8	43	Master of Nursing	20	14
RN9	42	Master of Nursing	20	17
RN10	52	Bachelor of Nursing	30	18
RN11	42	Bachelor of Nursing	20	20
RN12	44	Master of Nursing	22	22
RN13	49	Bachelor of Nursing	28	14
RN14	53	Bachelor of Nursing	30	21
RN15	47	Bachelor of Nursing	25	25
Mean	47.93	—	26	20.27

**Table 2:** Help-based assistance to elderly patients ill with CVD who experience fear of death: categories and subcategories.

Category	Subcategory	Interview excerpts
Help-based assistance	Communicating empathetically	*I try to calm patients down, not show fear on my face* (RN3).
		*I try to listen and hear the patient, calm and encourage* (RN6).
		*I establish trusting relationships with patients and their relatives* (RN8).
		*I communicate patiently, express interest in the patient*'*s problems, create a comfortable environment* (RN12).
		*I take into account the patient's individual needs, adapting communication to meet their unique needs* (RN14).
	Providing information	*I try to provide information on issues of concern to the patient* (RN5).
		*If necessary, I invite psychologists to the department to provide the necessary information* (RN9).
		*I provide information about the disease process, I do not use medical terms so that the patient does not have any ambiguities* (RN11).
		*I help patients discuss their pain and fears* (RN12).
	Creating a therapeutic atmosphere	*I use art and music as therapeutic tools to help with relaxation* (RN7).
		*I teach patients relaxation and breathing exercises* (RN11).
		*I provide comfort to the patient, use meditation* (RN15).

**Table 3:** Providing support to elderly patients ill with CVD who experience fear of death and their families: categories and subcategories.

Category	Subcategory	Interview excerpts
Providing support	Recommending therapy for emotional well-being	*I recommend various therapeutic measures, art or music therapy* (S2).
		*I offer spiritual classes if the person feels the need for spiritual support* (S4).
		*If the patient is creative, I encourage them to participate in artistic activities* (S6).
		*I recommend various therapeutic measures, breathing and meditation exercises* (S11).
		*I encourage them to engage in creative activities* (S13).
	Organising special professional assistance	*I suggest the patient seek help from a psychologist or therapist* (S3).
		*I teach the patient not to be afraid to discuss their problems and concerns with the doctor* (S7).
		*I encourage patients to exercise, and specialist help can also be used* (S9).
	Encouraging family involvement	*I recommend that family members participate more actively in the nursing process* (S11).
		*I encourage family members to engage in joint activities with the patient, to participate in the nursing process* (S13).
		*I teach family members meditation and breathing exercises together with patients* (S15).

**Table 4:** Emotions experienced by nurses while working with elderly patients ill with CVD who experience fear of death: categories and subcategories.

Category	Subcategories	Interview excerpts
Living with emotions	Acting with compassionate mercy	*I experience compassion for patients who are facing death* (RN2).
		*I believe that compassion is a natural reaction to other people*'*s suffering* (RN6).
		*I feel compassion for the patient*'*s suffering and experiences, I think that pain and death are wrong* (RN9).
	Worrying about powerlessness	*I am worried and feel helpless about the health, emotional state, and experiences of patients* (RN2).
		*I often feel helpless when I cannot help patients* (RN8).
		*I feel helpless when I cannot reduce the suffering of a patient* (RN10).
	Feeling internal moral outrage	*I sometimes feel angry about the situation and the deaths of patients* (RN3).
		*I feel angry when I see a young person die* (RN5).
	Suffering from doubts	*I am tormented by doubts about what I am doing to help patients* (RN1).
		*Sometimes I doubt my actions, whether I am really doing everything correctly* (RN6).

**Table 5:** Managing personal emotions through self-help among nurses while working with elderly patients ill with CVD who experience fear of death: categories and subcategories.

Category	Subcategory	Interview excerpts
Managing own emotions	Maintaining professional distance	*I try to distance myself from feelings and emotions, not to get too attached to patients* (RN8).
		*I have been working for a long time, so I have learnt to manage my emotions and maintain a professional distance* (RN11).
		*I try to maintain a professional distance at work so that the patient can express his emotions* (RN13).
	Reflecting in- and after-acting	*I use reflection to manage my emotions, I reflect on my experiences, feelings, and actions* (RN5).
		*I write down the thoughts that arise in my diary, I can read them later, and draw conclusions* (RN9).
		*I constantly reflect, I think about my emotions, their impact on the work I do* (RN12).
	Detaching emotions from work	*I separate my emotions from the duties I perform, thus maintaining emotional health* (RN8).
		*I set high professional standards for myself, set goals and achieve them, thus limiting myself from emotions* (RN12).
	Making time for a hobby	*I dedicate free time to myself and interesting activities, thus maintaining stability* (RN15).

## Data Availability

The data that support the findings of this study are available on request from the corresponding author. The data are not publicly available due to privacy or ethical restrictions.
